# Artificially sweetened beverages do not influence metabolic risk factors: a systematic review and meta-analysis

**DOI:** 10.3389/fnut.2025.1482719

**Published:** 2025-05-09

**Authors:** Lina Qin, Yunfeng Yu, Rong Yu

**Affiliations:** ^1^The First Hospital of Hunan University of Chinese Medicine, Changsha, China; ^2^First Clinical College of Traditional Chinese Medicine, Hunan University of Traditional Chinese Medicine, Changsha, China

**Keywords:** artificially sweetened beverages, metabolic risk factors, change of weight, non-communicable diseases, systematic review

## Abstract

**Introduction:**

The influence of artificially sweetened beverages (ASBs) on metabolic risk factors for non-communicable diseases (NCDs) remains unclear. This study aimed to systematically review the literature concerning whether the effects of ASBs on body weight and metabolic risk factors are equivalent to those of unsweetened beverages (USBs).

**Methods:**

We searched PubMed, Embase, Web of Science, and Cochrane Library databases from their establishment until March 3, 2025. Only randomized controlled trials (RCTs) comparing ASBs and USBs were included. Literature screening, data extraction, and bias evaluations were performed. Statistical analyses were performed using Review Manager 5.4 and Stata 15.0 software.

**Results:**

Nine RCTs involving 1,457 individuals were included. Meta-analysis findings indicated no statistically significant differences between ASB and USB groups in terms of weight, waist circumference, fasting blood glucose, glycated hemoglobin, homeostatic model assessment for insulin resistance, total cholesterol, triglycerides, high-density lipoprotein cholesterol, low-density lipoprotein cholesterol, systolic blood pressure, and diastolic blood pressure (all *p* > 0.05).

**Conclusion:**

The study findings do not support the hypothesis that ASBs pose significant risks or benefits in terms of metabolic risk factors for NCDs. However, given this study applied a heterogeneous ASB formula, it could not adequately consider the role of specific artificial sweeteners. Further research is needed to evaluate the potential effect of different artificial sweeteners and their doses on health.

**Systematic review registration:**

https://www.crd.york.ac.uk, identifier CRD420251027794.

## 1 Introduction

Non-communicable diseases (NCDs) are a worldwide public health challenge that cause the deaths of approximately 41 million people annually, accounting for approximately 74% of global deaths (1). Metabolic risk factors, such as overweight/obesity, elevated blood pressure, elevated blood glucose, and dyslipidemia, are the major factors driving this burden, with elevated blood pressure alone implicated in 19% of global mortality (1), Compounding this crisis, hyperglycemia contributes to 20% of cardiovascular deaths (2), while obesity underlies 40% of metabolic-related fatalities (3). Sugar-sweetened beverages (SSBs) are major contributors of excessive dietary sugar and caloric intake and a common risk factor for metabolic abnormalities (1, 4, 5), SSBs are closely associated with weight gain, type 2 diabetes mellitus (T2DM), and an increased risk of cardiovascular disease (6, 7). Therefore, the American Academy of Nutrition and Dietetics advocates substituting SSBs with artificially sweetened beverages (ASBs), in dietary interventions to reduce consumption while maintaining palatability (8).

In Europe, approximately 33.2% of people report daily consumption of ASBs, especially those with a high body mass index (BMI) or those following a weight loss plan (9). ASB consumption among children in the United States almost doubled from 8.7% in 1999–2000 to 14.9% in 2007–2008 (10). While ASBs contain little added sugar and provide minimal energy, emerging observational studies have raised concerns about their potential effects. Some studies have shown that ASBs significantly increase the risk of overweight/obesity, T2DM, and metabolic syndrome (11, 12); however, other studies have reported that ASBs are not associated with metabolic diseases or even reduced the risk of metabolic diseases. Palmer et al. (13) conducted a prospective survey of 5,900 African American women and reported no significant association between ASB intake and T2DM incidence (14). These findings suggest that the role of ASBs in weight management and metabolic health remains controversial. Therefore, this systematic review and meta-analysis aimed to evaluate the potential effects of ASBs on metabolic outcomes compared with unsweetened beverages (USBs), with the goal of providing additional evidence to resolve this controversy.

## 2 Materials and methods

This study followed the PRISMA guidelines for systematic review and meta-analysis.

### 2.1 Literature search

PubMed, Embase, Web of Science, and Cochrane Library electronic databases were searched for studies that compared ASBs with USBs and reported metabolic-related concerns. The retrieval time limit ranged from database establishment to March 03, 2025. The retrieval method primarily involved using subject headings and free-text terms, with adaptive adjustments made based on the characteristics of each database. For example, search expressions for PubMed included “artificially sweetened beverage” OR “diet drink” OR “non-nutritive sweetened beverages” OR “low calorie sweetened beverages” OR “non-caloric soft drink,” with a filter applied for randomized controlled trials (RCTs). A detailed search strategy is presented in Supplementary Table 1. Relevant references and systematic reviews were searched manually to ensure as comprehensive a search as possible and to supplement the study.

### 2.2 Study selection

The inclusion criteria comprised the following: (i) study type: RCTs; (ii) research participants: healthy individuals or those with pre-existing metabolic diseases; (iii) intervention measures: low-calorie or non-calorie soft drinks with added artificial sweeteners consumed by the experimental (ASB) group (types of artificial sweeteners were not limited), while the control group (USB group) received any beverage without sweeteners such as drinking water, tea, or sparkling water; (iv) duration of the intervention: ≥ 6 months; and (v) outcome measures: body weight, waist circumference (WC), fasting plasma glucose (FPG), glycated hemoglobin (HbA1c), homeostatic model assessment for insulin resistance (HOMA-IR), total cholesterol (TC), triglycerides (TG), high-density lipoprotein cholesterol (HDL-C), low-density lipoprotein cholesterol (LDL-C), systolic blood pressure (SBP), diastolic blood pressure (DBP), and energy intake (EI).

The exclusion criteria comprised the following: (i) duplicate studies, (ii) studies in which raw data required for this analysis could not be obtained, and (iii) studies that did not report any of the outcomes required for quantitative synthesis in this meta-analysis.

### 2.3 Data extraction

Two researchers performed the literature screening and data extraction. Study titles and abstracts were first reviewed to eliminate duplicates and irrelevant articles. Potentially relevant articles were then downloaded and thoroughly reviewed to exclude studies that did not meet our inclusion criteria. Data extraction included general study characteristics (author, year, country, and sample size), participant characteristics (age, sex, health status, BMI, and duration of follow-up), and intervention details. Finally, the two researchers cross-checked the extracted data, and any disagreements were resolved through discussion or, if necessary, by consulting a third researcher for a final decision.

### 2.4 Bias risk assessment

Cochrane’s RevMan 5.4 software was used for risk assessment. Bias was evaluated in seven fields: generation of random sequence, distribution hiding, blindness of participants and staff, integrity of result data, selective reporting, and other biases. Each evaluation was graded as low-risk, uncertain, and high-risk, with the methodological quality of each study evaluated independently. In case of disputes, the two authors discussed their evaluations and reached a resolution.

### 2.5 Data integration and statistical analysis

For studies that included multiple groups, we extracted data from ASB and USB groups only. We extracted the mean difference and standard deviation (SD) in relation to differences between baseline and the last visit. For studies that did not directly provide an SD, we referred to the treatment method of Koch et al. (15). In accordance with the Cochrane Handbook Chapter 6.5.2.2 and 6.5.2.3, (16) we converted the standard error (SE) and 95% confidence intervals (CIs) using the following formulas:


(1) $$\begin{array}{c} {}[\mathrm{SD}=\mathrm{SE}\;\times \;\sqrt{n\;\text{or }\mathrm{SD}=}\\ \surd n\times (\text{upper limit}-\text{lower limit})/3.92] \end{array}$$

If baseline and last follow-up index data were reported without the SD of the difference, we according to the Cochrane Handbook Chapter 6.5.2.8 to estimate it, (16) using a correlation coefficient of 0.50, as follows:


(2) $$\begin{array}{c} {\mathrm{SD}}_{E,\;\text{change}}=\sqrt{{\mathrm{SD}}_{E,\;\text{baseline}}^{2}\;+}\\ \sqrt{{\mathrm{SD}}_{E,\;\text{final}}^{2}\;-\;(2\;\times \;0.50\;\times \;{\mathrm{SD}}_{E,\;\text{baseline}}\;\times \;{\mathrm{SD}}_{E,\;\text{final}})} \end{array}$$

For studies that provided multiple analysis sets, we used complete datasets.

Revman 5.4 software was first used for the meta-analysis. We employed the weighted mean difference (MD) as the effect index for measurement data of the same unit and the standardized mean difference (SMD) as the effect index for measurement data of different units. *I*^2^ was utilized to evaluate heterogeneity among the included studies, and an appropriate effect model was selected according to heterogeneity. If heterogeneity among the included studies was small (*I*^2^ < 50%), the fixed-effect model was adopted. Conversely, if statistical heterogeneity was significant (*I*^2^ ≥ 50%), the source of heterogeneity was analyzed and, after excluding influencing factors, the corresponding model was used for analysis. All indicators are presented as MDs or SMDs and 95% CIs. Furthermore, we used Egger’s test in Stata 15 software to evaluate publication bias. If the *p*-value was < 0.05, the difference was considered statistically significant.

We then used the leave-one-out method for the sensitivity analysis to check the robustness of the combined results when each study was excluded. Finally, the GRADE online tool was used to evaluate the certainty of the evidence, including the risk of bias, indirectness, inconsistency, inaccuracy, and publication bias.

## 3 Results

### 3.1 Literature retrieval

**FIGURE 1:**
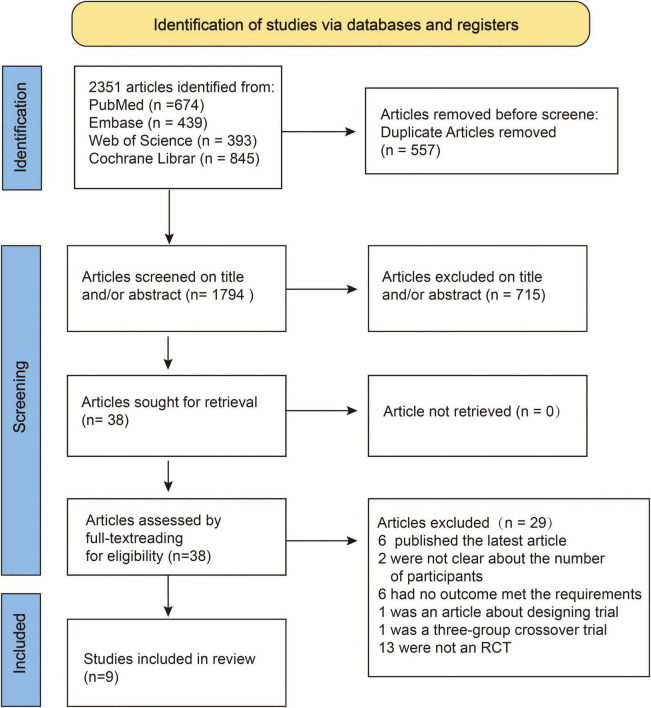
Flowchart of systematic literature review and selection process.

A preliminary search resulted in 2,351 articles, of which 38 were included in the full-text review. Finally, nine articles that met the criteria were included in the study. The screening process is illustrated in Figure 1.

### 3.2 Baseline study characteristics

The characteristics of the studies included in the meta-analysis are presented in Table 1. Nine studies were included (17–25), with a total baseline sample of 1,457 participants (women, *n* = 1,104; men, *n* = 353). Two studies included women only (20, 25). One study involved patients who were overweight or obese and had T2DM (25), six studies involved patients who were overweight or obese but did not have T2DM (18–23), and two studies involved participants of any weight (17, 24). Three studies were conducted in the United States (17, 22, 23), two were conducted in Denmark (18, 21) two were conducted in Iran (20, 25), one was conducted in the United Kingdom (19), and one was conducted in Mexico (24). The follow-up periods ranged from 24 to 77 weeks, with all studies following up the patients for at least 6 months. Of the nine studies, two (17, 23) used Equation 1 for data conversion, while two others (18 20) applied Equation 2. The remaining five studies retained original data without conversion.

**TABLE 1:** Baseline characteristics of studies identified in the systematic literature review.

Study	Country	Population	Partici-pants	Sex (Men/Women)		Age, (mean ± SD), y		BMI (kg/m^2^)		Duration	Beverage type
				**ASB**	**USB**	**ASB**	**USB**	**ASB**	**USB**		
Ebbeling et al. (17)	USA	Any weight, non-T2DM	136	40/27	41/28	26.7 ± 5.7	27.9 ± 6.0	26.1 ± 5.2	26.6 ± 4.6	12 months	ASB: Unrestricted species USB: spring water, purified water, bubble water
Engel et al. (18)	Denmark	Obese/ overweigh, non-T2DM	31	3/12	5/11	39.0 ± 7.6	39 ± 7.3	33.4 ± 1.1	31.5 ± 1.1	6 months	ASB: sugar-free coca-cola. USB: mineral water
Harrold et al. (19)	Britain	Healthy, BMI: 27–35 kg/m^2^	493	67/180	81/165	44.7 ± 12.0	46.0 ± 11.2	31.3 ± 2.2	31.3 ± 2.3	12 weeks of intervention+40 weeks of maintenane	ASB and water can be carbonated or still.
Madjd et al. (20)	Iran	Women, obese/ overweigh, non-T2DM	71	0/36	0/35	31.7 ± 6.8	32.2 ± 6.9	33.9 ± 3	33.9 ± 3	24 weeks of intervention+53 weeks of maintenane	ASB: any kind of ASB. USB: only allowing water
Maersk et al. (21)	Denmark	BMI: 26–40 kg/m^2^, non-T2DM	25	3/9	5/8	39 ± 8	39 ± 8	32.8 ± 3.8	32.2 ± 4.6	6 months	ASB: sugar-free coca-cola. USB: mineral water
Peters et al. (22)	USA	Healthy, BMI : 27–40 kg/m^2^	308	28/130	25/125	48.3 ± 10.4	47.3 ± 10.6	33.92 ± 4.25	33.30 ± 3.98	12 weeks of intervention+40 weeks of maintenane	ASB or bottled water from coca-cola or Pepsi group
Tate et al. (23)	USA	Healthy, BMI: 25–49.9 kg/m^2^	213	23/82	12/96	41.2 ± 11.2	43.2 ± 10.6	36.1 ± 6.2	35.8 ± 5.2	6 months	ASB: Any non-calorie sweet drink. USB: bottled water or non-sweet foaming water.
Vázquez et al. (24)	Mexico	Any weight, non-T2DM	99	10/40	10/39	21.46 ± 0.31	22.55 ± 0.51	25.48 ± 0.62	27.27 ± 0.70	6 months	ASB: only beverages with non-caloric sweeteners and sugar-free beverages were permitted. USB: no sweetened and sugar-free beverages were permitted.
Madjd et al. (25)	Iran	Obese/overweight, with T2DM	81	0/40	0/41	35.45 ± 7.45	34.15 ± 6.99	33.19 ± 2.25	32.86 ± 1.67	24 weeks	ASB: any kind of ASB. USB: only allowing water

ASB, artificially sweetened beverages; USB, unsweetened beverages; SSB, sugar-sweetened beverages; T2DM, type 2 diabetes mellitus. All 9 studies were included in the meta-analyses.

### 3.3 Risk assessment of bias

**FIGURE 2:**
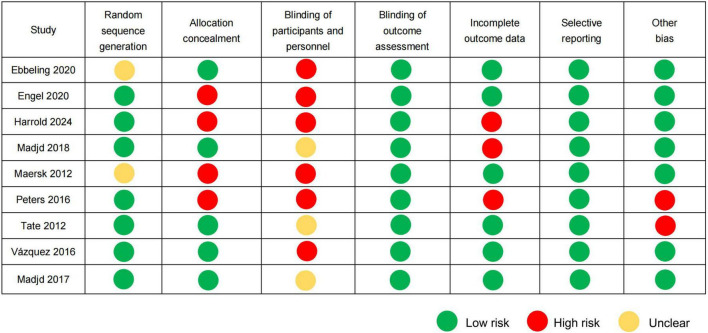
Risk of bias assessment according to the Cochrane guidelines.

Using random sequence generation through computerized randomization protocols, seven RCTs were classified as low-risk (18–20, 22–25), and two RCTs received an unclear risk designation because of insufficient documentation concerning the randomization methodology (17, 21). Five RCTs were rated as low-risk in allocation concealment because of the clear description of allocation concealment procedures (17, 20, 23–25), while four RCTs lacked sufficient methodological details, resulting in a high-risk of bias (18, 19, 21, 22). Three RCTs were classified as having an unclear risk in terms of blinding participants and personnel owing to inadequate blinding (20, 23, 25), and six RCTs were classified as high-risk because of an open design (17–19, 21, 22, 24). All nine RCTs reported objective biochemical indicators that were inherently unaffected by the subjective judgment of assessors. Consequently, these studies were deemed to have a low-risk of bias in the blinding domain of the outcome assessment. Three RCTs reported elevated dropout rates of 21.13, 27, and 46.86%, respectively, and were consequently rated as having a high risk for incomplete outcome data (19, 20, 22). The remaining six RCTs maintained dropout rates < 20% and were thus assessed as low-risk in this domain. All nine RCTs were assessed as having a low-risk of selective outcome reporting bias owing to the absence of evidence indicating discrepancies between pre-specified and reported outcomes. Two RCTs were funded by beverage companies and were assessed as high-risk with other biases (22, 23). The risk assessment in relation to bias is shown in Figure 2.

### 3.4 Meta-analysis results

#### 3.4.1 Weight and WC analysis

**FIGURE 3:**
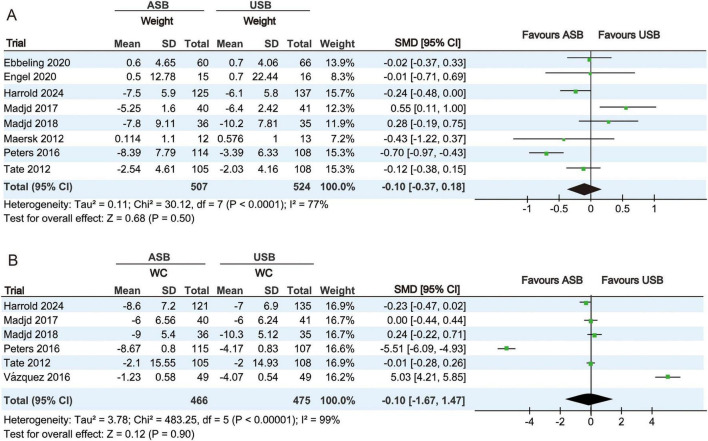
Forest plots illustrating changes in body weight and WC between the ASB and USB groups. **(A,B)** Body weight and WC Squares represent effect estimates within each study, with 95% CIs represented by horizontal lines. Square size is proportional to the weight of each study. Diamonds indicate the weighted mean effect estimates. ASB, artificially sweetened beverage groups; CI, confidence interval; SMD, standardized mean difference; USB, unsweetened beverage groups; WC, waist circumference.

Body weight at baseline and the last follow-up were measured in eight RCTs (17–23, 25). Owing to significant heterogeneity (*p* < 0.001, *I*^2^ = 77%), a random-effect model was used for the meta-analysis. No significant differences in body weight changes were observed between the ASB and USB groups (SMD, −0.10; 95% CI, −0.37 to 0.18; *p* = 0.50) (Figure 3A).

WC measurements at baseline and last follow-up were measured in six RCTs (19, 20, 22–25). Owing to significant heterogeneity (*p* < 0.001, I^2^ = 99%), a random-effects model was used for the meta-analysis. No significant differences in WC changes were observed between the ASB and USB groups (SMD, −0.10; 95% CI, −1.67 to 1.47; *p* = 0.90) (Figure 3B).

#### 3.4.2 Glucose metabolism index analysis

**FIGURE 4:**
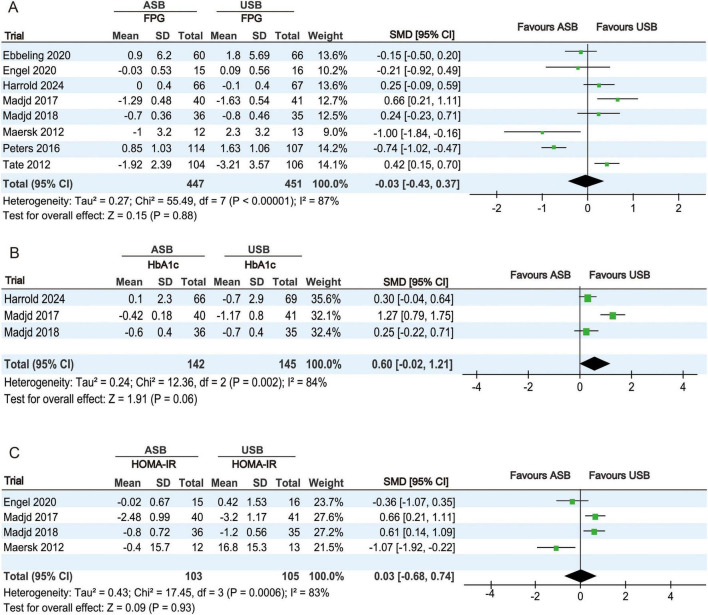
Forest plots displaying changes in glucose metabolism-related indicators between the ASB and USB groups. **(A)** FPG, **(B)** HbA1c, and **(C)** HOMA-IR. Squares represent effect estimates within each study, with 95% CIs represented by horizontal lines. Square size is proportional to the weight of each study. Diamonds represent the weighted mean effect estimates. ASB, artificially sweetened beverage groups; CI, confidence interval; FPG, fasting plasma glucose; HbA1c, glycated hemoglobin; HOMA-IR, homeostatic model assessment for insulin resistance; SMD, standardized mean difference; USB, unsweetened beverage groups.

The FPG levels at baseline and at the last follow-up were reported in eight RCTs (17–23, 25). Owing to significant heterogeneity (*p* < 0.001, *I*^2^ = 87%), a random-effects model was used for the meta-analysis. No significant differences in FPG levels were observed between the ASB and USB groups (SMD, −0.03; 95% CI, −0.43 to 0.37; *p* = 0.88) (Figure 4A).

HbA1c percentages at baseline and at the last follow-up were reported in three RCTs (19, 20, 25). Owing to significant heterogeneity (*p* = 0.002, *I*^2^ = 84%), a random-effects model was used for the meta-analysis. No significant differences in HbA1c changes were observed between the ASB and USB groups (SMD, 0.60; 95% CI, −0.02 to 1.21; *p* = 0.06) (Figure 4B).

HOMA-IR values at baseline and at the last follow-up were calculated in four RCTs (18, 20, 21, 25). Owing to significant heterogeneity (*p* < 0.001, *I*^2^ = 83%), a random-effects model was used for the meta-analysis. No significant differences in HOMA-IR changes were observed between the ASB and USB groups (SMD, 0.03; 95% CI, −0.68 to 0.74; *p* = 0.93) (Figure 4C).

#### 3.4.3 Analysis of lipid metabolism indexes

**FIGURE 5:**
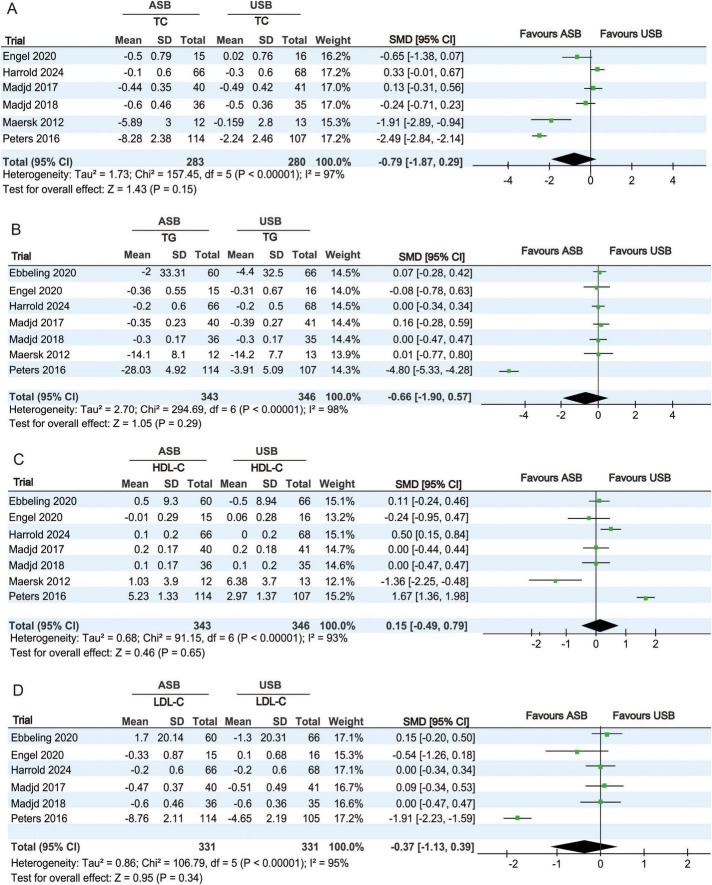
Forest plots showing differences in lipid metabolism indicators between the ASB and USB groups. **(A)** TC, **(B)** TG, **(C)** HDL-C, and **(D)** LDL-C. Squares indicate effect estimates within each study, with 95% CIs represented by horizontal lines. Square size is proportional to the weight of each study. Diamonds signify the weighted mean effect estimates. ASB, artificially sweetened beverage groups; CI, confidence interval; HDL-C, high-density lipoprotein cholesterol; LDL-C, low-density lipoprotein cholesterol; SMD, standardized mean difference; TC, total cholesterol; TG, triglycerides; USB, unsweetened beverage groups.

TC levels at baseline and at the last follow-up were recorded in six RCTs (18–22, 25). Owing to significant heterogeneity (*p* < 0.001, *I*^2^ = 97%), a random-effects model was used for the meta-analysis. No significant differences in TC changes were observed between the ASB and USB groups (SMD, −0.79; 95% CI −1.87 to 0.29; *p* = 0.15) (Figure 5A).

TG levels at baseline and at the last follow-up were recorded in seven RCTs (17–22, 25). Owing to significant heterogeneity (*p* < 0.001, *I*^2^ = 98%), a random-effects model was used for the meta-analysis. No significant differences in TG changes were observed between the ASB and USB groups (SMD, −0.66; 95% CI, −1.90 to 0.57; *p* = 0.29) (Figure 5B).

HDL-C levels at baseline and at the last follow-up were recorded in seven RCTs (17–22, 25). Owing to significant heterogeneity (*p* < 0.001, *I*^2^ = 93%), a random-effects model was used for the meta-analysis. No significant differences in HDL-C changes were observed between the ASB and USB groups (SMD, 0.15; 95% CI, −0.49 to 0.79; *p* = 0.65) (Figure 5C).

LDL-C levels at baseline and at the last follow-up were recorded in six RCTs (17–20, 22, 25). Owing to significant heterogeneity (*p* < 0.001, *I*^2^ = 95%), a random-effects model was used for the meta-analysis. No significant differences in LDL-C changes were observed between the ASB and USB groups (SMD, −0.37; 95% CI, −1.13 to 0.39; *p* = 0.34) (Figure 5D).

#### 3.4.4 Blood pressure analysis

**FIGURE 6:**
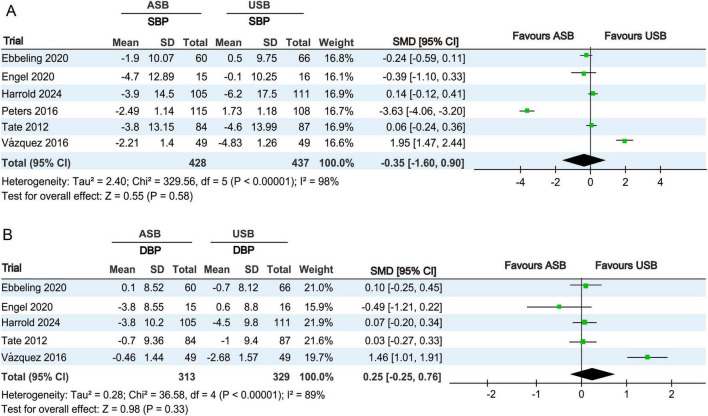
Forest plots revealing changes in blood pressures between the ASB and USB groups. **(A)** SBP and **(B)** DBP. Squares indicate effect estimates within each study, with 95% CIs represented by horizontal lines. Square size is proportional to the weight of each study. Diamonds represent the weighted mean effect estimates. ASB, artificially sweetened beverage groups; CI, confidence interval; DBP, diastolic blood pressure; SBP, systolic blood pressure; SMD, standardized mean difference; USB, unsweetened beverage groups.

SBP levels at baseline and at the last follow-up were measured in six RCTs (17–19, 21, 22, 24). Owing to significant differences (*p* < 0.001, *I*^2^ = 98%), a random-effects model was used for the meta-analysis. No significant differences in SBP changes were observed between the ASB and USB groups (SMD, −0.35; 95% CI, −1.60 to 0.90; *p* = 0.58) (Figure 6A).

DBP levels at baseline and last follow-up were measured in five RCTs (17–19, 21, 24). Owing to significant differences (*p* < 0.001, *I*^2^ = 89%), a random-effects model was used for the meta-analysis. No significant differences in DBP changes were observed between the ASB and USB groups (SMD, 0.25; 95% CI, −0.25 to 0.76; *p* = 0.33) (Figure 6B).

#### 3.4.5 EI analysis

**FIGURE 7:**
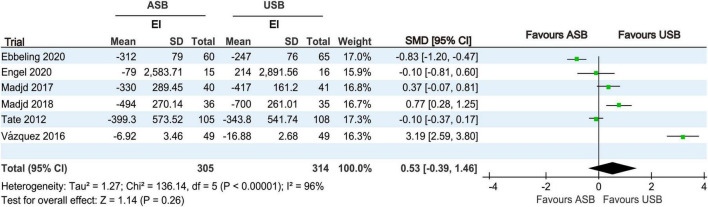
Forest plot displaying changes in EI between the ASB and USB groups. Squares represent effect estimates within each study, with 95% CIs represented by horizontal lines. Square size is proportional to the weight of each study. Diamonds represent the weighted mean effect estimates. ASB, artificially sweetened beverage groups; CI, confidence interval; EI, energy intake; SMD, standardized mean difference; USB, unsweetened beverage groups.

EI at baseline and at the last follow-up was recorded in six RCTs (17, 18, 20, 21, 24, 25). Owing to significant differences (*p* < 0.001, *I*^2^ = 96%), a random-effects model was used for the meta-analysis. No significant differences in EI changes were observed between the ASB and USB groups (SMD, 0.53; 95% CI, −0.39 to 1.46; *p* = 0.26) (Figure 7).

### 3.5 Meta-regression analysis

We conducted a meta-regression analysis to investigate the sources of heterogeneity in the meta-analysis. Owing to limited studies and excessive covariates risking overfitting, meta-regression was restricted to outcomes with ≥ 5 studies, focusing on three key variables (female proportion, behavioral guidance frequency, and follow-up duration), with other factors (ASB type, dose, age, BMI) being excluded. Our findings indicated that the behavioral guidance frequency significantly contributed to heterogeneity in both WC (*p* = 0.003) and HDL-C (*p* = 0.016). Furthermore, female participant proportion emerged as an additional significant modifier of HDL-C heterogeneity (*p* = 0.048). In addition, meta-regression analysis findings indicated that heterogeneity in weight, FPG, HbA1c, TC, TG, LDL-C, SBP, DBP, HOMA-IR, and EI could not be explained by female proportion, behavioral guidance frequency, and follow-up duration.

### 3.6 Sensitivity analysis

To further investigate the heterogeneity of a single source and evaluate the robustness of the results, we conducted sensitivity analysis using the leave-one-out method. First, the results showed that heterogeneity in terms of body weight, LDL-C and TG levels derived from an RCT by Peters et al. (22). After excluding Peters et al.’s (22) RCT, heterogeneity in terms of weight (*p* = 0.05, *I*^2^ = 52%), LDL-C (*p* = 0.57, *I*^2^ = 0%), and TG (*p* = 0.99, *I*^2^ = 0%) was significantly reduced, and the fixed-effects model maintained negative findings in relation to LDL-C (MD, 0.00; 95% CI, −0.11 to 0.11; *p* = 0.96) and TG (MD, 0.01; 95% CI, −0.05 to 0.22; *p* = 0.07). Second, heterogeneity in terms of HbA1c was primarily attributable to a 2017 RCT conducted by Madjd et al. (25). After excluding this RCT, heterogeneity was rendered non-significant (*p* = 0.85, *I*^2^ = 0%). Fixed-effects model analysis maintained the null effect (MD, 0.31; 95% CI, −0.32 to 0.94; *p* = 0.33), confirming the stability of these results. Third, heterogeneity observed in terms of DBP outcomes stemmed from an RCT by Vázquez et al. (24). After excluding that RCT, heterogeneity was not significant (*p* = 0.48, *I*^2^ = 0%). A fixed-effects model analysis maintained the null effect (MD, 0.27; 95% CI, −0.29 to 1.83; *p* = 0.73), confirming the stability of the results.

Leave-one-out sensitivity analyses further demonstrated robust meta-analysis results for weight, WC, FPG, HbA1c, HOMA-IR, TC, TG, HDL-C, LDL-C, SBP, DBP, and EI.

### 3.7 Publication bias

**FIGURE 8:**
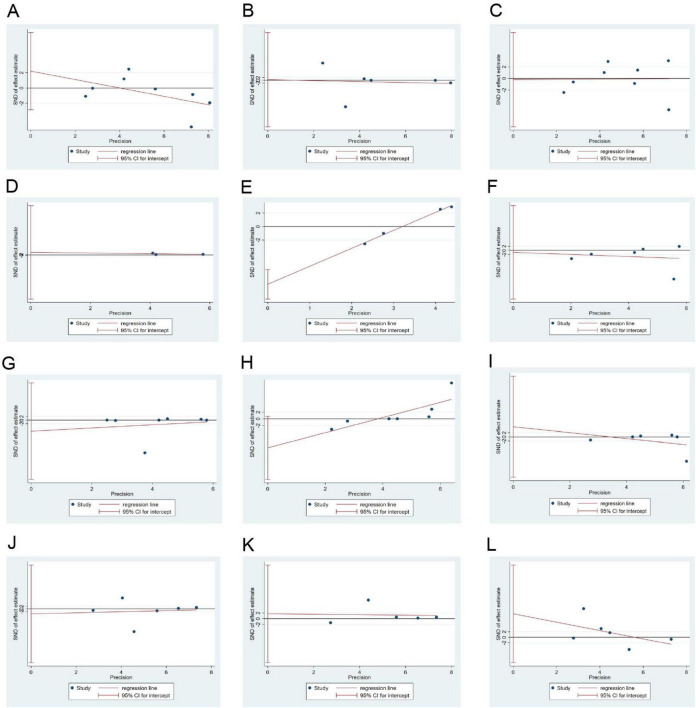
Egger’s test results for publication bias. **(A–L)**: Body weight, WC, FPG, HbA1c, HOMA-IR, TC, TG, HDL-C, LDL-C, SBP, DBP, and EI. DBP, diastolic blood pressure; EI, energy intake; FPG, fasting plasma glucose; HbA1c, glycated hemoglobin; HDL-C, high-density lipoprotein-cholesterol; HOMA-IR, homeostatic model assessment for insulin resistance; LDL-C, low-density lipoprotein-cholesterol; SBP, systolic blood pressure; TC, total cholesterol; TG, triglycerides; WC, waist circumference.

Except for the significant publication bias in relation to HOMA-IR (*p* = 0.004), publication biases in relation to items such as weight (*p* = 0.583), WC (*p* = 0.946), FPG (*p* = 0.951), HbA1c (*p* = 0.610), TC (*p* = 0.915), TG (*p* = 0.580), HDL-C (*p* = 0.064), LDL-C (*p* = 0.600), SBP (*p* = 0.790), DBP (*p* = 0.772), and EI (*p* = 0.815) were not significant (Figure 8).

### 3.8 Certainty of evidence

The GRADE Pro Evidence Quality Online Evaluation System was used to evaluate the certainty of the evidence, and the results showed that the evidence for weight and LDL-C was of low quality, whereas the evidence for WC, FPG, HbA1c, HOMA-IR, TC, TG, HDL-C, SBP, DBP, and EI was of very low quality (Table 2).

**TABLE 2:** Evaluation results of evidence quality.

Outcome index	No. of studies	Bias risk	Inconsistency	Indirectness	Imprecision	Publication bias	Effect value	Evidence grade
Weight	8	Serious	Not serious	Not serious	Not serious	Not serious	−0.10 [−0.37, 0.18]	Low
WC	6	Serious	Very serious	Not serious	Not serious	Not serious	−0.10 [−1.67, 1.47]	Very low
FPG	8	Serious	Serious	Not serious	Not serious	Not serious	−0.03 [−0.43, 0.37]	Very low
HbA1c	3	Serious	Serious	Not serious	Not serious	Not serious	0.60 [−0.02, 1.21]	Very low
HOMA-IR	4	Serious	Serious	Not serious	Very serious	Serious	0.03 [−0.68, 0.74]	Very low
TC	6	Serious	Very serious	Not serious	Not serious	Not serious	−0.79 [−1.87, 0.29]	Very low
TG	7	Serious	Very serious	Not serious	Not serious	Not serious	−0.66 [−1.90, 0.57]	Very low
HDL-C	7	Serious	Very serious	Not serious	Not serious	Not serious	0.15 [−0.49, 0.79]	Very low
LDL-C	6	Serious	Not serious	Not serious	Not serious	Not serious	−0.37 [−1.13, 0.39]	Low
SBP	6	Serious	Very serious	Not serious	Not serious	Not serious	−0.35 [−1.60, 0.90]	Very low
DBP	5	Serious	Serious	Not serious	Not serious	Not serious	0.25 [−0.25, 0.76]	Very low
EI	6	Serious	Very serious	Not serious	Not serious	Not serious	0.53 [−0.39, 1.46]	Very low

WC, waist circumference; FPG, fasting plasma glucose; HbA1c, glycated hemoglobin; HOMA-IR, homeostatic model assessment for insulin resistance; TC, total cholesterol; TG, triglycerides; HDL-C, high density lipoprotein cholesterol; LDL-C, low density lipoprotein cholesterol; SBP, systolic blood pressure; DBP, diastolic blood pressure.

## 4 Discussion

### 4.1 Research background and findings

The global burden of NCDs remains high, causing nearly three-quarters of all deaths worldwide each year (1). Reducing metabolic risk factors (e.g., being overweight, glucolipid metabolism disorders, and high blood pressure) can effectively prevent the occurrence of NCDs (26). Dietary glucose intake is considered to be related to obesity, diabetes, and cardiovascular diseases (27). Therefore, controlling dietary glucose intake has been key to the prevention and treatment of NCDs. Given that SSBs are a major source of dietary sugars, several systematic reviews have reported that replacing SSBs with ASBs can prevent body weight gain and reduce the risk of NCDs (28–30). Moreover, the American Heart Association has recommended using ASBs instead of SSBs (31). However, some studies have reported that ASBs may still increase the risk of metabolic diseases, such as T2DM and obesity (32, 33). Furthermore, because the energy balance of ASBs and SSBs is not well-established, the evidence remains inconclusive, and ASB consumption remains a controversial topic. To address this controversy, we conducted a meta-analysis to reassess the effects of ASBs and USBs on metabolism-related parameters. No significant difference was observed in relation to body weight, blood glucose, lipids, or blood pressure levels between the ASB and USB groups, indicating that ASBs have no significant effect on metabolic risk indicators.

### 4.2 Effects of ASBs on body weight and WC

Our meta-analysis showed similar effects concerning ASBs and USBs on body weight and WC measurements. Subsequent sensitivity analysis showed that the RCT by Peters et al. (22) introduced significant heterogeneity into the weight analysis, which was attributable to its different energy prescriptions and exercise plans compared with the other RCTs. Additionally, the meta regression analysis results suggested that heterogeneity in terms of WC was attributable to behavioral guidance frequency. Both body weight and WC exhibited heterogeneity, but the sensitivity analyses supported robustness in the related results. Moreover, among the included studies, four provided participants with lifestyle guidance, including dietary advice (19, 20, 23, 25). After excluding the relevant data from these four studies (19, 20, 23, 25), the significance of body weight and WC remained unchanged, indicating that differences between the ASB and USB groups were not influenced by weight-loss diets. Our findings are inconsistent with the conclusion of a 2016 meta-analysis published by Rogers et al. (14) in which greater weight loss was observed in the ASB group compared with the USB group. This discrepancy may stem from the limited number of studies in the meta-analysis by Rogers et al.’s (14), which included only three articles assessing body weight. Conversely, we included six additional RCTs published between 2016 and March 2025. Their analysis was largely influenced by a study by Peters et al. (34), which was based on an interim report published in 2014. Contrastingly, we included the most recent follow-up results from Peters et al. (22), including 12 weeks of intervention and a 40-week follow-up period. Therefore, we consider that our findings indicating similar effects of ASBs and SSBs on body weight is likely to be more reliable. Moreover, our meta-analysis confirmed that ASBs had no significant effect on WC, which is consistent with findings reported in the meta-analysis by McGlynn et al. (35). Thus, ASBs appear to have no significant effect on the maintenance of body weight and body shape.

### 4.3 Effect of ASBs on glycolipid metabolism

We observed no significant difference in HbA1c levels between the ASB and USB groups. The heterogeneity in HbA1c levels was primarily driven by a 2017 RCT by Madjd et al. (25), likely owing to the inclusion of a female-only T2DM population. Subsequent sensitivity analyses confirmed the robustness of this conclusion. However, a meta-analysis by McGlynn et al. (35) reported a significant improvement in HbA1c levels in a water consumption group. This discrepancy may be attributed to their inclusion of two short-term studies and the absence of published or updated studies after 2022. Furthermore, our meta-analysis showed no statistically significant differences in FPG and HOMA-IR between the ASB and USB groups. We did not find a source for any clinical and methodological heterogeneity in relation to FPG and HOMA-IR, but the sensitivity analyses confirmed the robustness of the results. In a crossover trial, Brown et al. (36) showed the comparable effects of ASBs and USBs on glucose metabolism. Following ASB or soda water consumption, oral glucose tolerance test results did not differ significantly in terms of blood glucose and C-peptide areas under the curve (AUC) from 10 min before to 180 min after glucose loading among healthy individuals and type 1 diabetes mellitus (T1DM) and T2DM cohorts (36). This conclusion was further supported by a 12-week RCT by Kendig et al. (37) and in a standardized dietary study by Atkinson et al. (38). Additionally, a meta-analysis by Zhang et al. (39) showed that the effects of single or mixed sweetener drinks on postprandial blood sugar and hormone secretion levels were similar to those of water, further supporting our findings. Therefore, for healthy individuals or T2DM populations, the effect of drinking ASBs in the short- and medium-term on blood sugar levels appears to be neutral.

Morevoer, ASBs had no significant effect on TC, TG, HDL-C, or LDL-C levels in this meta-analysis. Sensitivity analyses revealed that heterogeneity in terms of TG and LDL-C levels originated from the study conducted by Peters et al. (22), which can be attributable to its different energy prescriptions and exercise plans compared with other studies. Meta-regression analysis suggested that sex imbalance and behavioral guidance frequency significantly contributed to heterogeneity in relation to HDL-C levels. Most of the participants in these RCTs were females. However, we did not identify a heterogeneous source of TC. Our sensitivity analyses supported the robustness of the meta-analysis results for LDL-C, HDL-C, TC, and TG levels. This conclusion was supported by a multi-ethnic study on atherosclerosis, which found no statistically significant hazard ratios for high TG and low HDL-C among groups with varying ASBs consumption frequencies (12). Moreover, a meta-analysis published in 2022 (35) showed no significant differences in blood lipid levels between ASB and water groups, supporting our research results. In conclusion, ASBs were not shown to affect the levels of indicators related to lipid metabolism.

### 4.4 Effects of ASBs on blood pressure and energy metabolism

Our meta-analysis also showed that ASBs had no significant effect on SBP and DBP values. Heterogeneity in terms of DBP originated from a study by Vázquez et al. (24), whereas heterogeneity in terms of SBP could not be explained by clinical or methodological factors. The sensitivity analyses indicated that the meta-analysis results for DBP and SBP were robust. However, McGlynn et al. (35) reported that SBP was significantly reduced in an ASB group compared with a water group, whereas DBP values did not differ significantly between the groups. This inconsistency in relation to SBP can be attributable to differences in the inclusion criteria, as McGlynn et al. (35) included short-term studies with a follow-up period of < 6 months. Additionally, Kim and Je (40) reported that, compared with those who consumed almost no ASBs (< 0.8 parts/month), the relative risk of hypertension was increased by 9% in those who consumed ≥ 1 part of an ASB per day. Li et al. (41) reported similar results and identified a significant linear correlation between ASBs and hypertension. Their reports were based on prospective cohort studies, whereas we only included RCTs.

Finally, we evaluated the effect of ASBs on energy metabolism and observed no statistical difference between ASBs and USBs in terms of EI. This finding is consistent with that of a meta-analysis by Rogers et al. (14), which included a large number of animal experiments. Moreover, in a study by Creze et al. (42), after consuming ASBs and water, no significant difference was observed in spontaneous food intake among healthy men. Electroencephalography monitoring revealed that ASB intake did not affect the activity of the insula but increased neural activity in the ventrolateral prefrontal area, which is related to reward inhibition. Similarly, Fantino et al. (43) reported no difference between water and ASBs in terms of EI, macronutrient intake, and motivational ratings.

### 4.5 Important insights and significance

Our findings indicated that ASBs do not disrupt body weight, glycolipid metabolism, energy metabolism, or blood pressure, supporting its safety. In recent years, commercially available ASBs have emerged as the primary consumption pattern for artificial sweeteners (44, 45). Metabolic risk outcomes typically require extended intervention periods to manifest measurable changes. However, most RCTs investigating isolated artificial sweeteners are limited to short-term interventions (< 6 months) (46–48). This evidence gap suggests that research focusing on ASBs may hold greater clinical relevance, owing to their alignment with real-world consumption patterns. Other studies have shown that artificial sweeteners do not increase metabolism-related risks, indirectly supporting our findings. For example, a 12-week RCT reported no significant effects of sustained sucralose consumption on sweet receptor expression, EI, or metabolic parameters (49). Overall, glucose tolerance was not significantly affected by artificial sweeteners, although weight changes in one sucralose group were negative, whereas the intake of saccharin led to weight gain (46). In healthy adults, neither aspartame nor stevia impair glucose tolerance (48). While this evidence indicates that artificial sweeteners do not cause significant metabolic damage, their specific effects require further evaluation in future meta-analyses.

### 4.6 Limitations and prospects

This study had certain limitations. Owing to dietary and exercise guidance provided in four RCTs, those participants were more likely to lose weight and improve their metabolism than in daily life, which limits the explanatory power of the research conclusions. The populations included in this meta-analysis were diverse, and the intervention methods varied, leading to significant heterogeneity. This limits the evidential weight of our conclusions. The RCTs included in this meta-analysis involved follow up for ≥ 6 months. However, determining clear changes in relation to metabolic diseases requires several years of observation. The number and scale of the included RCTs were relatively limited; therefore, the conclusions warrant further research support. The funding sources may raise concerns. Two of nine included studies had received financial support from beverage companies—a potential conflict of interest that may have biased the results in favor of ASBs (50). In our meta-analysis, the proportion of female participants was high (75.8%), which may limit the generalizability of our conclusions to male individuals. Finally, this meta-analysis focused only on the effects of ASBs on the metabolic risk factors for NCDs; it did not consider the role of a single artificial sweetener. We look forward to additional high-quality RCTs that can facilitate further improved understanding of the role and effects of ASBs.

## 5 Conclusion

ASB consumption showed no significant association with adverse alterations in metabolic risk markers for NCDs. However, as this study was based on a heterogeneous ASB formula, it did not address the role and effect of a single artificial sweetener. Further research is needed to evaluate the potential effect of different artificial sweeteners and their doses on health.

## Data Availability

The original contributions presented in this study are included in this article/Supplementary material, further inquiries can be directed to the corresponding author.
