# Supplementary material for: Artificially sweetened beverages do not influence metabolic risk factors: a systematic review and meta-analysis
Source: Front Nutr. 2025 May 9;12:1482719. doi: 10.3389/fnut.2025.1482719 (PMC12098100; doi:10.3389/fnut.2025.1482719)
Supplement: Supplementary file 1 [file Table_1.docx]

**Table S1.** Search terms and results for the systematic review.

| Database | Search terms | Filter | Results Yielded |
| --- | --- | --- | --- |
| Pubmed | artificially sweetened beverage OR diet Drink OR non-nutritive sweetened beverage OR low-calorie sweetened beverage OR non-caloric soft drink | Randomized Controlled Trial | 674 |
| Embase | 'artificially sweetened beverage'/exp OR 'artificially sweetened beverages'/exp OR 'diet drink'/exp OR 'non-nutritive sweetened beverages’/exp or ‘low-calorie sweetened beverages’/exp or ‘non-caloric soft drink'/exp | - | 439 |
| Web of Science | TS=(artificially sweetened Beverage OR diet drink OR non-nutritive sweetened beverage OR low-calorie sweetened beverage OR non-caloric soft drink) AND TS=(water OR Unsweetened Beverage) AND TS=(metaboli* OR blood sugar OR blood pressure OR lipids or energy) AND TS=( randomized controlled trial) | - | 393 |
| Cochrane Library | (artificially Sweetened Beverage OR diet Drink OR non-nutritive sweetened beverage OR low-calorie sweetened beverage OR non-caloric soft drink) AND (randomized controlled trial OR randomized clinical trial OR RCT) | - | 845 |
| Total | | | 2351 |
